# Supplementary material for: Chronic Oleoylethanolamide Treatment Decreases Hepatic Triacylglycerol Level in Rat Liver by a PPARγ/SREBP-Mediated Suppression of Fatty Acid and Triacylglycerol Synthesis
Source: Nutrients. 2021 Jan 27;13(2):394. doi: 10.3390/nu13020394 (PMC7910994; doi:10.3390/nu13020394)
Supplement: Supplementary file 1 [file nutrients-13-00394-s001.pdf]

Supplementary Materials:

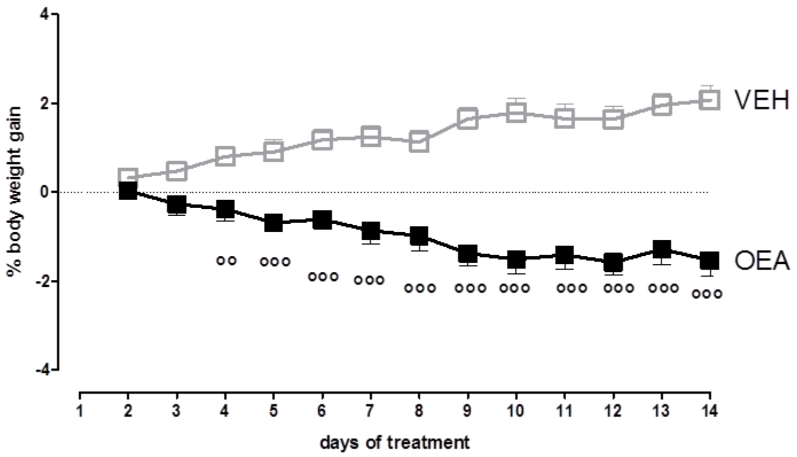

Figure S1. Rats daily administered with OEA 10 mg kg^{-1} i.p. (black squares) showed a significantly lower body weight gain with respect to VEH treated rats (open gray squares). Such difference was significant starting from the fourth day of treatment and lasted for the rest of the treatment period. Statistical analysis was performed by two way ANOVA for repeated measures, followed by Bonferroni's post hoc test for multiple comparisons. Data are expressed as means ± SEM; °°p<0.01, °°°p<0.001 vs respective VEH; N=12.

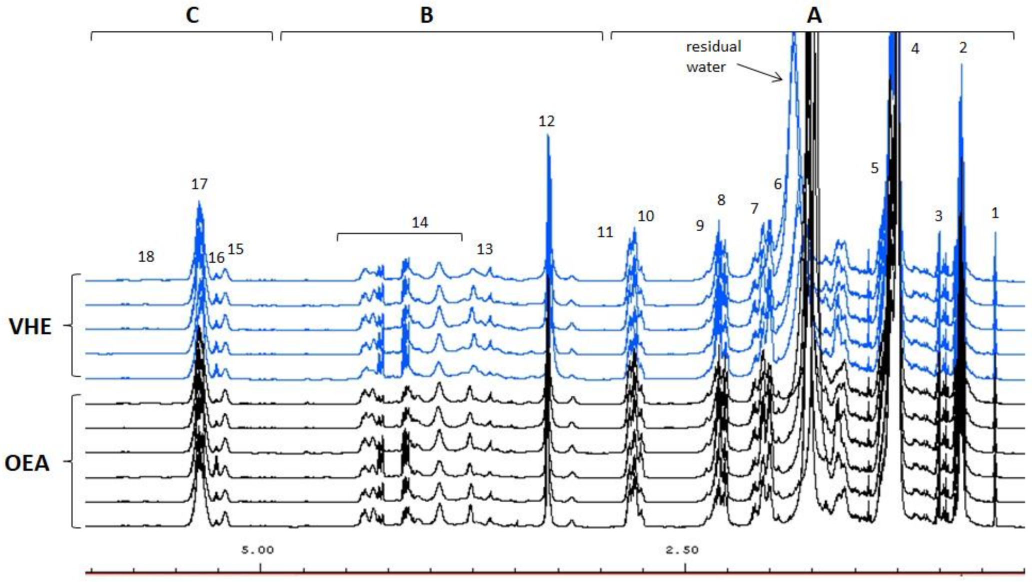

Figure S2. ^{1}H NMR spectra of lipid extracts with identified NMR signals (region **A**: 3.00-0.65 ppm, typical of fatty acids and sterol methyl and methylene resonances; region **B**: 5.00-3.00 ppm for phospholipids head groups and glycerol backbone proton resonances; region **C**: 6.00-5.00 ppm for vinyl protons resonances). Numbers are referred to the corresponding signal assignment reported in Table 1.

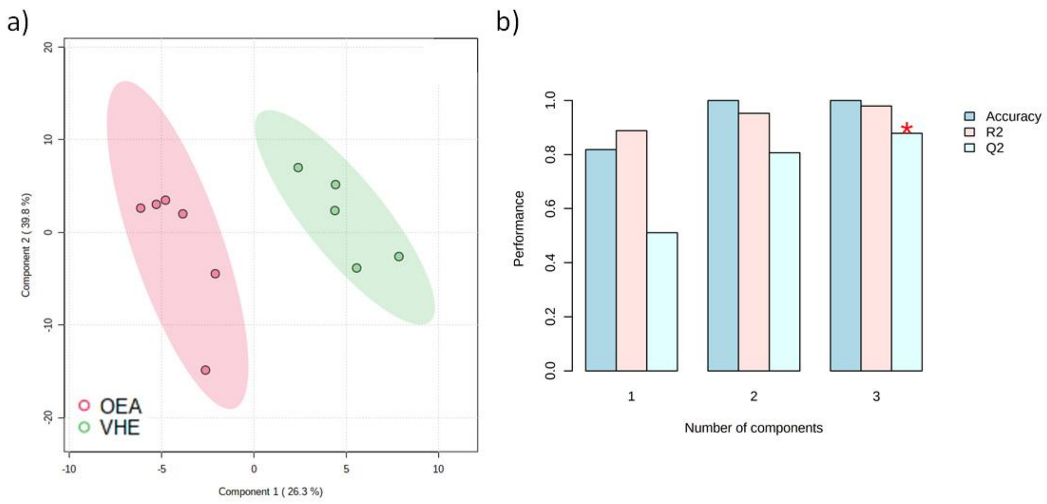

Figure S3. (**a**) Partial least squares discriminant analysis (PLS-DA) score plot and **(b)** cross validation details (10-fold), in terms of R^2 and Q^2 for OEA and VEH samples.

Table S1. MS/MS operating conditions. Multiple reaction monitoring (MRM) functions and settings for detection of sphingolipids are shown. Sa: Sphinganine (d18:0); d 17:0 Sa: Sphinganine (d17:0); So: Sphingosine (d18:1); d 17:1 So: Sphingosine (d17:1); Sa1P: Sphinganine-1-phosphate (d18:0); d 17:0 Sa1P: Sphinganine-1-phosphate (d17:0); So1P: Sphingosine-1-phosphate (d18:1); d 17:1 So1P: Sphingosine-1-phosphate (d17:1); C16Cer: C16:0 Ceramide (d18:1/16:0); C17Cer: C17 Ceramide (d18:1/17:0); C18Cer: C18 Ceramide (d18:1/18:0); C16dHCer: C16 Dihydroceramide (d18:0/16:0); C16GlcCer: C16 Glucosyl(ß) Ceramide (d18:1/16:0); C17GlcCer: C17 Glucosyl(ß) Ceramide (d18:1/17:0); C22Cer:C22 Ceramide (d18:1/22:0); C24Cer: C24 Ceramide (d18:1/24:0); C24dHCer: C24 Dihydroceramide (d18:0/24:0).

MRM Function	Time window (min)	Analyte	Transitions (*m/z*)	Cone Volts	Coll Energy (eV)
**1**	3-7	Sa	302.3 > 284.2	14	50
		d 17:0 Sa	288.2 >270.2	14	
**2**	3-7	So	300.2 > 282.2	10	50
		d 17:1 So	286.2 > 268.2	11	
**3**	4-8.5	Sa1P	382.2 > 284.2	13	50
		d 17:0 Sa1P	368.2 > 270.2	13	
**4**	4.5-10	So1P	380.2 > 264.2	15	50
		d 17:1 So1P	366.2 > 250.2	15	
**5**	7-13.5	C16Cer	538.4 > 264.2	19	50
		C17Cer	552.5 > 264.2	18	
		C18Cer	566.5 > 264.2	21	
		C16dHCer	540.5 > 522.5	18	
		C16GlcCer	700.5 > 520.4	15	
		C17GlcCer	714.6 > 264.2	18	
**6**	12-20	C22Cer	622.5 > 264.2	23	50
		C24Cer	650.6 > 264.2	22	
		C24dHCer	652.5 > 634.7	23	
